# Sr-Containing Mesoporous Bioactive Glasses Bio-Functionalized with Recombinant ICOS-Fc: An In Vitro Study

**DOI:** 10.3390/nano11020321

**Published:** 2021-01-27

**Authors:** Sonia Fiorilli, Mattia Pagani, Elena Boggio, Casimiro Luca Gigliotti, Chiara Dianzani, Rémy Gauthier, Carlotta Pontremoli, Giorgia Montalbano, Umberto Dianzani, Chiara Vitale-Brovarone

**Affiliations:** 1 Department of Applied Science and Technology, Politecnico di Torino, Corso Duca degli Abruzzi 24, 10129 Torino, Italy; mattia.pagani@polito.it (M.P.); remy.gauthier@polito.it (R.G.); carlotta.pontremoli@polito.it (C.P.); giorgia.montalbano@polito.it (G.M.); chiara.vitale@polito.it (C.V.-B.); 2 NOVAICOS s.r.l.s, Via Amico Canobio 4/6, 28100 Novara, Italy; elena.boggio@med.uniupo.it (E.B.); luca.gigliotti@med.uniupo.it (C.L.G.); 3 Department of Health Sciences, Università del Piemonte Orientale, Via Solaroli 17, 28100 Novara, Italy; umberto.dianzani@med.uniupo.it; 4 Department of Drug Science and Technology, Università di Torino, Via Pietro Giuria 9, 10125 Torino, Italy; chiara.dianzani@unito.it

**Keywords:** mesoporous bioactive glasses, nano-biomaterials, strontium release, osteoporosis, ICOS-L, bone remodeling

## Abstract

Osteoporotic bone fractures represent a critical clinical issue and require personalized and specific treatments in order to stimulate compromised bone tissue regeneration. In this clinical context, the development of smart nano-biomaterials able to synergistically combine chemical and biological cues to exert specific therapeutic effects (i.e., pro-osteogenic, anti-clastogenic) can allow the design of effective medical solutions. With this aim, in this work, strontium-containing mesoporous bioactive glasses (MBGs) were bio-functionalized with ICOS-Fc, a molecule able to reversibly inhibit osteoclast activity by binding the respective ligand (ICOS-L) and to induce a decrease of bone resorption activity. N_2_ adsorption analysis and FT-IR spectroscopy were used to assess the successful grafting of ICOS-Fc on the surface of Sr-containing MBGs, which were also proved to retain the peculiar ability to release osteogenic strontium ions and an excellent bioactivity after functionalization. An ELISA-like assay allowed to confirm that grafted ICOS-Fc molecules were able to bind ICOS-L (the ICOS binding ligand) and to investigate the stability of the amide binding to hydrolysis in aqueous environment up to 21 days. In analogy to the free form of the molecule, the inhibitory effect of grafted ICOS-Fc on cell migratory activity was demonstrated by using ICOSL positive cell lines and the ability to inhibit osteoclast differentiation and function was confirmed by monitoring the differentiation of monocyte-derived osteoclasts (MDOCs), which revealed a strong inhibitory effect, also proven by the downregulation of osteoclast differentiation genes. The obtained results showed that the combination of ICOS-Fc with the intrinsic properties of Sr-containing MBGs represents a very promising approach to design personalized solutions for patients affected by compromised bone remodeling (i.e., osteoporosis fractures).

## 1. Introduction

Under normal clinical conditions, the bone tissue shows a potential of self-healing after injury [1]. Despite this intrinsic regenerative efficacy, still up to 10–15% of the fractures show a delayed or non-union situations [1,2], which could depend on a variety of causes, including the patient clinical situation and the occurrence of concomitant complications, such as bacterial infections. Moreover, with the increase of elderly population and the associated pathological conditions such as osteoporosis, the total number of unsuccessful bone healing cases is expected to dramatically increase in the next future [3]. The occurrence of osteoporosis is due to an imbalance between osteoclast bone resorption and osteoblast bone formation and exposes the affected people to a high increased risk of fracture, which currently represents a critical clinical issue and requires the adoption of new and personalized treatments. In order to stimulate bone tissue regeneration in the elderly population, one of the most interesting approach is the design of multifunctional nano-biomaterials enriched with chemical and biological cues, able to concurrently attain several effects, including osteoconductivity and the ability to stimulate osteogenesis and angiogenesis [4,5,6,7,8]. In the field of bone tissue engineering, mesoporous bioactive glasses (MBGs) have gained increasing attention for their enhanced bioactivity and their ability to release therapeutic species, proving successful outcomes in stimulating bone regeneration. In fact, the extremely high exposed surface area and regular nanoporosities allow to vehicle a variety of active molecules, including anti-inflammatory [9,10] and antimicrobial agents, proteins and growth factors [11]. Moreover, according to the final application, MBG composition can be enriched with specific elements (i.e., Sr, Cu, Ag [6,12,13]) to impart related therapeutic effects. Among the investigated elements, strontium has attracted considerable attention due to its well-known role in the activation of both osteoblastic and osteoclastic cell signaling pathways, which allows the promotion of osteoblast replication, differentiation, and survival while downregulating osteoclast activities [14]. However, the specific effect on osteoclast maturation and activity is reported to be strictly dependent on the strontium administrate doses, as sub-effective concentrations did not reveal the ability to inhibit osteoclast-mediated bone resorption [15].

The successful incorporation of strontium into MBG framework has been previously investigated by the authors [16] who demonstrated the effective pro-osteogenic role of released Sr^2+^ ions, along with the excellent biocompatibility of the material. In addition, with the rationale to widen the therapeutic potential, Sr-Cu substituted MBGs have been developed [17], where the pro-osteogenic effect of strontium was combined with the ability to promote neovascularization and with the anti-microbial action exerted by copper ions [9,12,18]. Besides the enrichment of the composition with elements acting as chemical cues, the conjugation of MBG surface with molecules exerting a biological effect is straightforward thanks to the high number of surface hydroxyl groups, which allows an easy functionalization through the consolidated and versatile alkoxysilane chemistry [19,20]. By following this route, biological molecules, such as growth factors, peptides, or antibodies can be easily anchored to the MBG surface to further improve the overall tissue regeneration potential [21,22].

In this contribution with the aim to open new perspectives for an advanced and personalized treatment of osteoporotic fractures, Sr-containing MBGs, already widely proved in the literature to exert an in vitro pro-osteogenic effect [23] have been functionalized with ICOS-Fc, a recombinant molecule able to reversibly inhibit osteoclast activity as described in a patent (WO/2016/189428) by the authors [24]. More in detail, ICOS is a T cell co-stimulatory surface receptor, which binds to ICOS-L, a surface receptor expressed by several cell types, including osteoclasts. Recent findings have shown that the binding ICOS:ICOS-L is involved in the regulation of bone turnover and that the administration of a soluble recombinant form of ICOS (ICOS-Fc) reversibly inhibits human osteoclast activity in vitro [25]. Moreover the authors demonstrated that the administration of ICOS-Fc in vivo is able to inhibit and possibly reverse the development of experimental osteoporosis (OP) in mice [25].

**Figure 1:**
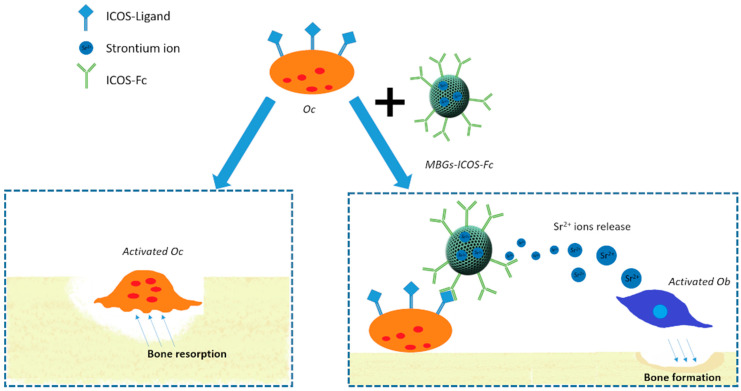
Schematic illustration of Sr-containing mesoporous bioactive glasses (MBGs) functionalized with ICOS-Fc on osteoblast and osteoclast cells.

The potential breakthrough of these findings represents the motivation underlying this contribution which aims at the synergistic integration of ICOS-Fc biological effect with the release of pro-osteogenic Sr^2+^ ions and the excellent bioactivity of MBGs, as schematically depicted in Figure 1, with the ambition to face the challenges of stimulating bone tissue regeneration in the elders.

The proposed strategy would allow us to combine for the first time the intrinsic properties of MBGs with the specific effect exerted by ICOS-Fc to deliver a multifunctional (i.e., bioactivity, stimulation of osteoblast cells, inhibition of osteoclast activity) and versatile platform for the design of new and personalized medical solutions.

With this final goal in mind, Sr-containing MBGs (10 mol%) were produced by using two different procedures, a base-catalyzed sol-gel method and an aerosol-assisted spray-drying approach, in order to obtain nano-sized and micro-sized particles respectively, with different morphological and structural features. In order to provide MBG surface with reactive amines able to covalently bind the carboxylic groups exposed on Fc residue of ICOS-Fc, a post-synthesis functionalization step with (3-aminopropyl)silanetriol (APST) has been optimized, prior the molecule grafting by exploiting the EDC/NHS chemistry.

Bio-functionalized materials were fully characterized to assess the effective anchoring of ICOS-FC and the complete retention of the intrinsic MBG features in terms of bioactivity and ion release, since grafting could potentially hinder or even suppress surface ion-exchange reactions. To this aim, strontium ion release and in vitro bioactivity experiments were conducted on ICOS-Fc grafted MBGs and the binding stability in aqueous medium was monitored up to 21 days.

In view of the envisaged final application, a comprehensive in vitro biological assessment was conducted to demonstrate the biocompatibility of the developed bio-functionalized materials and the proper accessibility and orientation of ICOS-Fc at the surface, allowing the retention of its biological activity. To this aim, the effect on cell proliferation and the ability to impair the migration of ICOSL positive cells were investigated and compared to free ICOS-Fc.

Furthermore, since the reported effect of ICOS-Fc on bone metabolism is due to the inhibition of osteoclast differentiation and function, the retention of this effect by grafted ICOS-Fc is an essential feature. This aspect was investigated by assessing the ability of bio-functionalized materials to inhibit the differentiation of human monocyte-derived osteoclasts in comparison to free ICOS-Fc.

## 2. Materials and Methods

### 2.1. Preparation of MBGs Containing 10 mol% of Strontium (MBGs-Sr)

MBGs-Sr were prepared using two different synthesis approaches, a base-catalyzed sol–gel synthesis and an aerosol-assisted spray-drying method resulting in nano- and microparticles respectively and different features in terms of specific surface area, pore volume, and nanopore size.

#### 2.1.1. Sol-Gel (SG) Synthesis of Sr-Containing MBGs (SG-Sr)

SG-Sr were prepared by modifying a procedure optimized by the authors [16]. In brief, 12 g of cetyltrimethylammonium bromide (CTAB 98%, Sigma Aldrich, Italy) were dissolved in 140 mL of ethanol, 50 mL of double distilled water (ddH_2_O) and 25 mL of NH_4_OH (Ammonium hydroxide solution, Sigma Aldrich, Italy) for 30 min under stirring. A second solution containing 9.4 g of tetraethyl orthosilicate (TEOS, Tetraethyl orthosilicate, reagent grade 98%, Sigma Aldrich, Italy) and 50 mL of ethanol was prepared and stirred for 30 min. Then, the TEOS solution was added dropwise into CTAB solution and the resulting solution was stirred for 20 min. 0.94 g of calcium nitrate tetrahydrate (Ca(NO_3_)_2_·4H_2_O, 99%, Sigma Aldrich, Italy) and 1.69 g of strontium nitrate (Sr(NO_3_)_2_, 99%, Sigma Aldrich, Italy) were added and stirred for 10 min. A further amount of TEOS (4.7 g) was then added drop by drop to the solution and stirred for 2 h. The powder was collected by centrifugation (Hermle Labortechnik Z326) at 10,000 rpm for 3 min and washed three times with ddH_2_O, once with 50% ethanol and once with absolute ethanol. The final precipitate was dried at 70 °C overnight and calcined at 600 °C for 5 h with a heating rate of 1 °C min^−1^ in a Carbolite 1300 CWF 15/5, in order to remove CTAB. All the reagents were purchased from Sigma Aldrich (Italy) and used as received.

#### 2.1.2. Spray-Dried (SD) Synthesis of Sr-Containing MBGs (SD-Sr)

SD-Sr were prepared using a spray-drying method, by modifying the procedure reported by Pontremoli et al. [19]. Briefly, 2.03 g of the non-ionic block copolymer Pluronic P123 (EO_20_PO_70_EO_20_, average M_n_ ~ 5800, Sigma Aldrich, Milan, Italy) were dissolved in 85 mL of ddH_2_O until the solution appeared transparent (about 3 h). In a separate batch, TEOS (10.73 g) was pre-hydrolyzed in acidic conditions with 5 g of an aqueous solution of HCl (pH 2) until the solution was transparent (about 3 h). Then, the solution of TEOS was added dropwise to the P123 solution. To the resulting solution were then added 1.62 g of strontium chloride (SrCl_2_ 99%, Sigma Aldrich, Milan, Italy) and 0.72 g of calcium nitrate tetrahydrate. The resulting solution was then sprayed (Büchi, Flawil, Switzerland, Mini Spray-Dryer B-290) using nitrogen as the atomizing gas (inlet temperature 220 °C, N_2_ pressure 60 mmHg, feed rate 5 mL min^−1^). Finally, the obtained powder was calcined at 600 °C for 5 h with a heating rate of 1 °C min^−1^ in a Carbolite 1300 CWF 15/5 to remove the surfactant. All the reagents were purchased from Sigma Aldrich (Milan, Italy) and used as received.

### 2.2. Grafting of ICOS-Fc Molecule on Aminated MBG Surface

The extracellular portion of human ICOS was cloned as fusion protein to the human IgG1 Fc region, generating ICOS-Fc fusion protein, according to Di Niro et al. [26]. In particular, after transfection, cells stably and highly expressing ICOS-Fc were established. The human ICOS-Fc fusion protein was harvested and purified from supernatant by protein A affinity column.

**Figure 2:**
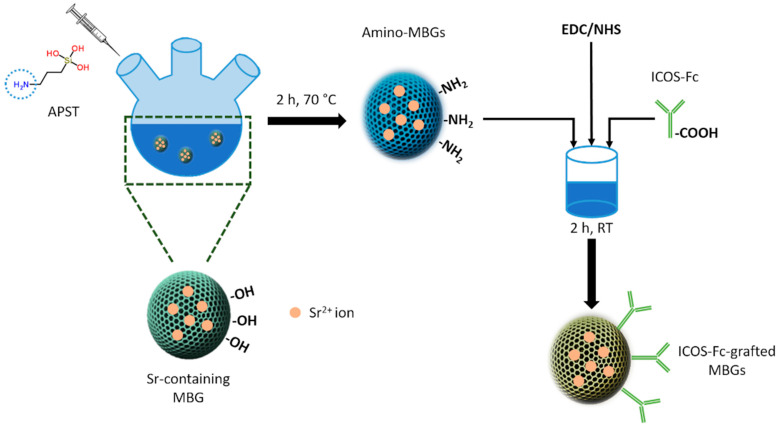
ICOS-Fc grafting on amino-MBGs surface.

The grafting of ICOS-Fc on MBG surface was achieved following a covalent zero-distance approach, as reported schematically in Figure 2. At first, strontium containing MBGs were functionalized to expose reactive amino groups on the surface and successively, ICOS-Fc was grafted exploiting the carboxyl groups present on Fc residue.

SG-Sr or SD-Sr was reacted with ((3-aminopropyl)silanetriol to obtain amino-functionalized MBGs (Amino-SG-Sr and Amino-SD-Sr, respectively) by applying the procedure generally reported for mesoporous silicas [19,27,28]. Briefly, 600 mg of MBGs were outgassed at room temperature and dispersed in 50 mL of ethanol under nitrogen atmosphere (Schlenk line). Then, 500 µL of APST were added to the suspension and the reaction was carried out for 2 h at 70 °C. The powder was then filtered and washed three times with ethanol. Finally, the product was dried overnight at 70 °C and collected. The obtained functionalized MBGs were used to bind ICOS-Fc molecule, following the procedure described by Wang et al. [29]. Briefly, 1 mg of ICOS-Fc was dissolved in 10 mL of phosphate-buffered saline (PBS). Then, 200 µL of a solution of 1-Ethyl-3-(3-dimethylaminopropyl)carbodiimide (EDC, 50 mg/mL) and N-Hydroxysuccinimide (NHS, 75 mg/mL) was added. The solution was stirred for 15 min at room temperature and then 500 mg of Amino-SG-Sr or Amino SD-Sr were added. The reaction was carried out for 2 h at room temperature. Finally, the product was collected, washed 5 times with PBS, and dried under vacuum overnight. All the reagents were purchased from Sigma Aldrich (Italy) and used as received.

### 2.3. Physico-Chemical Characterization

The morphology of the produced particles was analyzed by Field-Emission Scanning Electron Microscopy (FE-SEM) and the presence of strontium by Energy Dispersive X-ray Spectrometry (EDS) using a ZEISS MERLIN instrument. For FE-SEM observations, 10 mg of SG-Sr and SG-Sr-ICOS were dispersed in 10 mL of isopropanol using an ultrasonic bath (Digitec DT 103H, Bandelin, Berlin, Germany) for 5 min. The resulting suspension was dropped on a copper grid (3.05 mm Diam.200 MESH, TAAB, Berks, UK), allowed to dry and successively chromium-coated prior to imaging with a Cr layer of 7 nm. The SD-Sr and SD-Sr-ICOS powder, at variance, was dispersed directly to a conductive carbon tape adhered on a stub and coated with the 7 nm Cr layer.

To evaluate the effective amount of strontium incorporated into MBGs during the synthesis, the powders were dissolved in a mixture of nitric and hydrofluoric acids (0.5 mL of HNO_3_ and 2 mL of HF for 10 mg of powder) and the resulting solutions were measured via ICP analysis.

A size analysis was performed for SG-Sr and SG-Sr-ICOS-Fc using a Dynamic Light Scattering (DLS) method with a Zetasizer nano ZS90 (Malvern Instruments Ltd., Malvern, UK). The preparation consisted to disperse 3 mg of SG-Sr or SG-Sr-ICOS-Fc were in 3 mL of ddH2O to obtain a concentration of 1 mg/mL and sonicate for 10 min. Then, the solution was analyzed.

N_2_ adsorption-desorption measurements were conducted by using an ASAP2020 Micromeritics analyzer at a temperature of −196 °C. Before measurements, samples were outgassed at 150 °C for 5 h. The Brunauer–Emmett–Teller (BET) equation was used to calculate the specific surface area (SSA_BET_) from the adsorption isotherm in the 0.04–0.2 relative pressure range. The pore size distribution was calculated through the DFT method (Density Functional Theory) using the NLDFT kernel of equilibrium isotherms (desorption branch).

The presence of ICOS-Fc was assessed by Fourier Transform infrared spectroscopy (FT-IR) in transmission mode. The samples were compressed into a pellet and outgassed in vacuum for one hour at room temperature before FT-IR analysis. FT-IR spectra were collected using a FT-IR spectrometer (Bruker Equinox 55 spectrometer, Billerica, MA, USA) in the 4000–600 cm^−1^ wavenumber range. The successful grafting of ICOS-Fc was assessed by soaking 3 mg of SG-Sr-ICOS-Fc or SD-Sr-ICOS-Fc in Dulbecco’s modified Eagle’s medium (DMEM) (Sigma Aldrich) supplemented with 10% heat-inactivated fetal bovine serum (FBS) (Lonza BioWhittaker, Barcelona, Spain), 1 mM Lglutamine (Lonza BioWhittaker, Porriño, Spain), 200 mg/mL penicillin (Lonza BioWhittaker, Porriño, Spain) and 200 mg/mL streptomycin (Lonza BioWhittaker, Porriño, Spain) at room temperature for 3, 7, 14 and 21 days. At each time step, the amount of ICOS-Fc in the supernatant was analyzed by an ELISA-like assay, in order to evaluate the possible detachment from MBG surface due to the amide bond hydrolysis. The same assay was also conducted on the powder recovered at each time step.

### 2.4. In Vitro Bioactivity of SG-Sr-ICOS-Fc and SD-Sr-ICOS-Fc

The in vitro bioactivity test was performed in simulated body fluid (SBF) according to the protocol described in the literature [30], with the aim to evaluate the apatite-forming ability of the ICOS-Fc-grafted MBGs. In brief, 30 mg of SG-Sr-ICOS-Fc and SD-Sr-ICOS-Fc were soaked in 30 mL of SBF at 37 °C up to 14 days in an orbital shaker (Excella E24, Eppendorf, Milan, Italy) with an agitation rate of 150 rpm. At each time point (3 h, 1 day, 3 days, 7 days and 14 days), the suspension was centrifuged at 10,000 rpm for 5 min, the collected powder was washed twice with distilled water and dried in oven at 70 °C for 12 h prior FE-SEM, and X-ray diffraction (XRD) analysis to evaluate the apatite layer formation. XRD analysis were performed using a Philips X’Pert diffractometer with a Cu Kα (40 kV, 20 mA) source in a 2θ range of 10–70 with a scan speed of 0.05 °/s. Moreover, the pH of each recovered supernatant was measured to assess the suitability of the environment for maintaining the physiological activity of osteoblasts [31].

### 2.5. Sr^2+^ Release from SG-Sr-ICOS-Fc and SD-Sr-ICOS-Fc

The concentration of Sr^2+^ ions released from biofunctionalized MBGs was evaluated by soaking the powders in Tris HCl buffer (Tris(hydroxymethyl)aminomethane (Trizma) ((Sigma Aldrich, Milan, Italy) 0.1 M, pH 7.4) at concentration of 250 μg/mL, by following the procedure reported by the authors [16,19]. In particular, 5 mg of powder were suspended in 20 mL of buffer up to 14 days at 37 °C in an orbital shaker (Excella E24, Eppendorf) with an agitation rate of 150 rpm. At predefined time points (3 h, 24 h, 3 days, 7 days and 14 days) the suspension was centrifuged at 10,000 rpm for 5 min (Hermle Labortechnik Z326, Wehingen, Germany), half of the supernatant was collected and replaced by the same volume of fresh buffer solution to keep constant the volume of the release medium. The release experiments were carried out in triplicate. The concentration of Sr^2+^ ions was measured by Inductively Coupled Plasma Atomic Emission Spectrometry Technique (ICP-AES) (ICP-MS, Thermoscientific, Waltham, MA, USA, ICAP Q), after appropriate dilutions. The experiment was performed three times for each sample and the data are presented as means ± standard deviations.

### 2.6. Assessment of Grafted ICOS-Fc Functionality

#### 2.6.1. ELISA-Like Assay

ELISA plates were coated by overnight incubation at 4 °C with 1 μg/mL of human ICOSL-His (Sino-Biological, Beijing, China) in PBS 1X, pH 7.4. After 5 washes with 0.05% Tween-20 in PBS 1X (pH 7.4), non-specific binding was blocked by 1 h incubation with the same solution, at room temperature. After 1 h, samples were added in duplicate and incubated for 2 h at 37 °C. After 5 washes, horseradish peroxidase (HRP)-conjugated goat anti-human immunoglobulin (Ig)G (Dako, Santa Clara, CA, USA) was added and incubated for 1 h at room temperature. After 5 washes, TMB (tetra-methyl-benzidine) (Merck Life Science, Darmstadt, Germany) was added to each well and the reaction was stopped by adding H_2_SO_4_ 2N (Merck Life Science). The samples were analyzed using a plate reader spectrophotometer at 450 nm (Packard SpectraCount, Meriden, CT, USA). Results were recorded as optical density (OD).

The described assay was performed to measure: (i) the amount of residual ICOS-Fc in the supernatant recovered after the grafting reaction (unbound ICOS-Fc); (ii) the functionality of ICOS-Fc grafted on MBGs (functionality test); (iii) the binding stability of ICOS-Fc upon soaking in DMEM up to 21 days (stability test).

#### 2.6.2. Cytofluorimetric Assay

In order to check the presence of ICOS-Fc, SG-Sr-ICOS-Fc and SD-Sr-ICOS-Fc were also analyzed by means of flow cytometry (Attune NxT, Life Technologies, Carlsbad, CA, USA). Powders re-suspended in PBS 1X were saturated with 1% Normal Goat Serum (NGS) (R&D System, Minneapolis, MN, USA) + 1% Bovine Serum Albumin (BSA) (Merck Life Science) + 0.05% Tween-20 (Merck Life Science) for 20 min at 4 °C. After the indicated time, samples were centrifuged at 10,000 rpm for 5 min, re-suspended in PBS 1X+ 1% NGS and stained with an allophycocyanin (APC)-conjugated anti-human ICOS (R&D System) monoclonal antibody for 20 min at 4 °C. After washing, samples were fixed and analyzed with a flow cytometer. The mean fluorescence intensity ratio (MFI-R) was calculated dividing the MFI of stained samples (arbitrary units) by the MFI of the respective unstained sample (arbitrary units). The same analysis was conducted with SG-Sr and SD-Sr as blank samples.

### 2.7. Cells for Biocompatibility and Invasion Assays

The MC3T3-E1, murine pre-osteoblast, the PC-3 (ICOSL positive) from human prostate carcinoma, and the U2OS (ICOSL positive) human osteosarcoma cell lines were obtained from the American Type Culture Collection (Manassas, VA, USA). The HOS (ICOSL negative) human osteosarcoma cell line was obtained from Sigma-Aldrich. MC3T3-E1 and U2OS were grown as a monolayer in DMEM, PC-3 in RPMI 1640 (Gibco, Life Technologies, Carlsbad, CA, USA), HOS in MEM (Gibco) + 1% non-essential aminoacids (Sigma-Aldrich). All media were supplemented with 10% Fetal Bovine Serum (FBS), 100 U/mL penicillin, and 100 μg/mL streptomycin (Gibco) and cells were mainta ined at 37 °C in a 5% CO_2_ humidified atmosphere.

### 2.8. Biocompatibility of SG-Sr-ICOS-Fc and SD-Sr-ICOS-Fc

MC3T3-E1 cells were seeded in 96-well plates in complete DMEM medium (Gibco). After 24 h, the medium was removed and cells were incubated for 2, 4, and 7 days in the medium containing titrated amounts (200–100–10 μg/mL) of SG-Sr-ICOS-Fc and SD-Sr-ICOS-Fc. After the indicated times of incubation, viable cells were evaluated by adding XTT [2,3-Bis(2-methoxy-4-nitro-5-sulfophenyl)-2H-tetrazolium-5-carbox-anilide)] reagent (Trevigen, Helgerman CT, Gaithersburg, MD, USA) for 3 h at 37 °C. The samples were read by a plate reader spectrophotometer at 490 nm. Cell viability was calculated with the following formula:

$$\mathrm{cell}\;\mathrm{viability}\;=\;\frac{\mathrm{absorbance}\;\mathrm{of}\;\mathrm{sample}}{\mathrm{absorbance}\;\mathrm{of}\;\mathrm{control}\;\left(\mathrm{untreated}\;\mathrm{cells}\right)\;}\times \;100$$

Three independent experiments were performed. Statistical analyses were performed using GraphPad Prism 3.0 software (San Diego, CA, USA), as well as the one-way ANOVA and Dunnett Multiple Comparison tests. Values of *p* < 0.05 were considered statistically significant.

### 2.9. Cell Migration Assay with SG-Sr-ICOS-Fc and SD-Sr-ICOS-Fc

In the Boyden chamber (BD Biosciences, Milan, Italy) migration assay, 2 × 10^3^ PC-3 cells were plated onto the apical side of 50 μg/mL Matrigel-coated filters (8.2 mm diameter and 0.5 μm pore size; Neuro Probe, Inc.; BIOMAP snc, Milan, Italy) in serum-free medium with or without of ICOS-Fc grafted MBGs at different concentrations (2; 0.2; 0.02; 0.002 μg/mL). Medium containing 20% FBS was placed in the basolateral chamber as a chemoattractant. After 6 h, cells on the apical side were wiped off with Q-tips. The cells on the bottom of the filter were stained with crystal-violet and all counted with an inverted microscope. The data are shown as percentages of the inhibition of treated cells versus the control migration measured on untreated cells. Five independent experiments were performed. Statistical analyses were performed as reported for biocompatibility studies.

### 2.10. Clonogenic Assay

Osteosarcoma cells (5 × 10^3^ cells/well) were seeded into six-well plates. The day after, the cells were treated with different concentrations of SG-Sr-ICOS-Fc and SD-Sr-ICOS-Fc for 72 h. Then, the medium was changed, and the cells were cultured for additional 7 days in a MBGs-free medium. Subsequently, cells were fixed and stained with a solution of 80% crystal violet (Sigma–Aldrich) and 20% methanol and colonies were photographed. After that, the cells were washed, and 30% *v/v* acetic acid was added to induce a complete dissolution of the crystal violet. Absorbance was recorded at 595 nm by a 96-well-plate ELISA reader. Five different experiments were performed.

### 2.11. Monocyte-Derived Osteoclast (MDOCs) Differentiation and Culture

Peripheral Blood Mononuclear Cells (PBMCs) were separated from human blood samples obtained from buffy coats, provided by the local Blood Transfusion Service (Novara, Italy), by density gradient centrifugation using the Ficoll–Hypaque reagent (Lympholyte-H; Cedarlane Laboratories, Burlington, ON, Canada). The use of buffy coats was approved by the local ethical committee (n. CE 88/17, protocol 583/CE). Monocyte-derived Osteoclasts (MDOCs) were prepared from CD14^+^ monocytes isolated with the Easy Sep Human CD14 Negative Selection Kit (STEMCELL Technologies, Vancouver, BC, Canada). The monocytes were plated in 6-well (1 × 10^6^) or 24-well plates (0.2 × 10^6^) and cultured for 21 days in a differentiation medium composed of DMEM (Lonza, Basel, Switzerland), 2 mM of L-glutamine, 10% FBS (Invitrogen, Carlsbad, CA, USA), recombinant human M-CSF (25 ng/mL; R&D System, Minneapolis, MN, USA), and RANK-L (30 ng/mL; R&D System). The differentiation medium was changed every 3 days, and, at different times, cells were treated with free ICOS-Fc or with ICOS-Fc grafted Sr-containing MBGs (SG-Sr-ICOS-Fc or SD-Sr-ICOS-Fc) at 2 μg/mL. The same experiments were also conducted with bare SG-Sr or SD-Sr for comparison. Phase-contrast images were acquired by Axiovert 40 CFL microscope (Zeiss, Oberkochen, Germany) equipped with a Q imaging camera and Image Pro Plus 7.0 software (Media Cybernetics Inc, Rockville, MD, USA).

### 2.12. Real-Time RT-PCR

Total RNA was isolated from MDOCs cultures at day (T) T21 using TRIzol reagent (Invitrogen). RNA was retrotranscribed using the QuantiTect Reverse Transcription Kit (Qiagen, Hilden, Germany). DC-STAMP, OSCAR, and NFATc1 expression were evaluated with a gene expression assay (Assay-on Demand; Applied Biosystems, FosterCity, CA, USA). The GAPDH gene was used to normalize the cDNA amounts. Real-time PCR was performed using the CFX96 System (Bio-Rad Laboratories, Hercules, CA, USA) in duplicate for each sample in a 10 μL final volume containing 1 μL of diluted cDNA, 5 μL of TaqMan Universal PCR Master Mix (Applied Biosystems), and 0.5 μL of Assay-on Demand mix. The results were analyzed with the ΔΔ threshold cycle method.

### 2.13. TRAP Activity

Tartrate-resistant acid phosphatase (TRAP) activity was assessed using the Acid Phosphatase kit (Sigma-Aldrich) according to the manufacturer’s instructions. Briefly, cells were fixed with Citrate Solution 0.038 mol/L in 60% Acetone. After fixation cells were washed and incubated in a pre-warmed labeling solution (Fast Garnet GBC Base Solution 7 mg/mL dissolved in Acetate Solution 2.5 mol/L, pH 5.2, Naphtol AS-BI phosphoric acid solution 12.5 mg/mL, Tartrate Solution 0.67 mol/L, pH 5.2) for 1 h at 37 °C. Microphotographs of TRAP staining were acquired by EVOS FLoid Cell Imaging System (Life Technologies, Carlsbad, CA, USA).

## 3. Results and Discussion

### 3.1. Physico-Chemical Characterization of SG-Sr-ICOS-Fc and SD-Sr-ICOS-Fc

**Figure 3:**
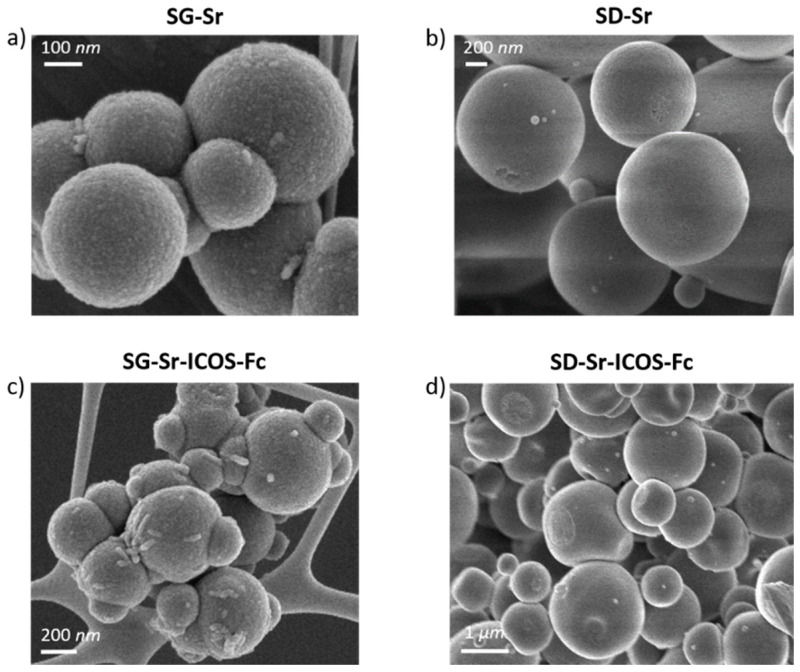
FE-SEM images of SG-Sr (**a**), SD-Sr (**b**), SG-Sr-ICOS-Fc (**c**), and SD-Sr-ICOS-Fc (**d**).

The FE-SEM observations showed a uniform spherical morphology and size both for bare and grafted particles. In particular, SG-Sr and SG-Sr-ICOS-Fc showed a size ranging between 100 and 500 nm, while SD-Sr and SD-Sr-ICOS-Fc size was measured to be between 0.5 and 5 μm, confirming previous results reported by the authors [16]. As shown in Figure 3, the morphological analysis revealed that grafting of ICOS-Fc did not lead to significant morphological variations of the particle size and morphology. DLS analyses confirmed the average diameter size of SG-Sr particles (Figure S1a) and evidenced, as expected, a larger diameter for SG-Sr-ICOS-Fc due to the presence of the biomolecule at the surface and related hydration sphere (Figure S1b).

EDS analysis was performed on SG-Sr-ICOS-Fc and SD-Sr-ICOS-Fc to evidence the presence of strontium incorporated into MBG framework and the related values are reported in Table S1.

Since the effective amount of strontium incorporated into MBGs during the synthesis cannot be precisely quantified by energy dispersive spectroscopy (EDS) due to an overlapping of the strontium and silicon signals, the chemical composition of SG-Sr and SD-Sr was determined by ICP-AES. The measurements revealed that particles contain 9.8 mol% and 7.9 mol% of strontium for SG-Sr-ICOS-Fc and SD-Sr-ICOS-Fc, respectively. These results confirmed the incorporation of strontium ions with a molar percentage close to the theoretical one. The slight lower amount encountered for SD-Sr-ICOS-Fc can be supposedly ascribed to the larger ionic radius of strontium (1.16 Å) compared to that of calcium (0.94 Å) [32], which can partially limit the incorporation into the framework during the spray-drying process, as previously observed by the authors [16].

ICP analysis on samples after the biofunctionalization evidenced the same amount of incorporated strontium, confirming that the two steps procedure to graft ICOS-Fc did not cause any significant loss of incorporated strontium during the procedure.

**Figure 4:**
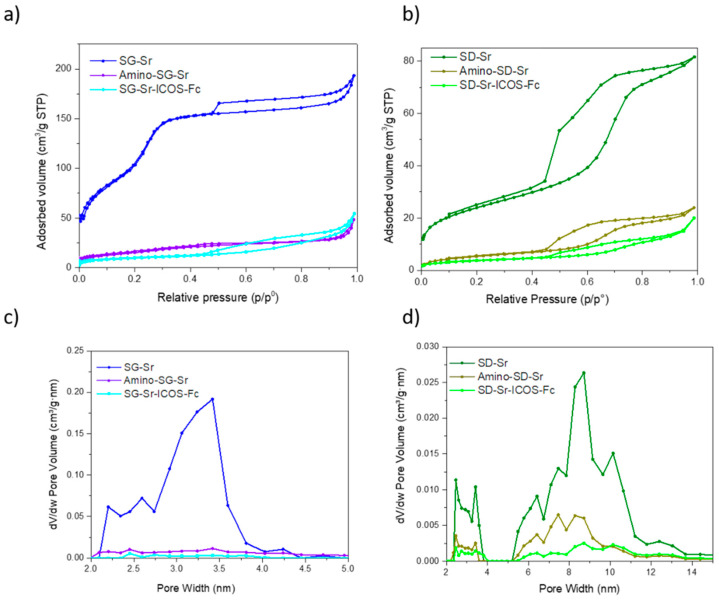
N_2_ adsorption-desorption isotherm of SG-Sr, Amino-SG_Sr and SG-Sr-ICOS-Fc (**a**), SD-Sr, Amino-SD_Sr and SD-Sr-ICOS-Fc (**b**). Pore size distribution of SG-Sr, Amino-SG_Sr and SG-Sr-ICOS-Fc (**c**), SD-Sr, Amino-SD_Sr, and SD-Sr-ICOS-Fc (**d**).

The textural properties were evaluated by N_2_ adsorption/desorption measurements to investigate specific surface area (SSA_BET_), pore volume and pore size of samples before and after functionalization. SG-Sr showed a type IV isotherm, typical of the mesoporous materials, and pore size distribution ranging between 2 and 4 nm (Figure 4a,c). The sample revealed high specific surface area equal to 465 m^2^/g and pore volume of 0.33 cm^3^/g (Table 1). N_2_ adsorption/desorption of SD-Sr showed a type IV isotherm (Figure 4b) with a pronounced hysteresis loop and a SSA of 85 m^2^/g, pore size in the range of 7–11 nm (Figure 4d) and pore volume of 0.13 cm^3^/g (Table 1).

SG-Sr-ICOS-Fc and SD-Sr-ICOS-Fc exhibited an evident modification of the isotherm and a drastic decrease in SSA and pore volume compared to the corresponding bare samples (Figure 4), suggesting that mesopores were almost fully blocked by amino groups and by ICOS-Fc molecules grafting. Due to the steric hindrance of ICOS-Fc, the latter is expected to mostly react with amino groups at the mesopore entrances during the initial phases of reaction, leading to partial or even full pore occlusion [19,20,33]. This is particularly evident for the SG-Sr sample, which, due to the smaller pore size compared to SD-Sr, shows almost full occlusion of the porosities after amines and ICOS-Fc grafting, as revealed by the isotherm modification (Figure 4a) and related pore size distribution (Figure 4c). The appearance of a more pronounced hysteresis after ICOS-Fc grafting (SG-Sr-ICOS-Fc) can be ascribed to the formation of nanocavities due to the biomolecule packing at the surface. As far as SD-Sr sample is concerned, a significant decrease of surface area and pore volume is also observed upon amino groups and ICOS-Fc grafting, however only a partial mesopore occlusion occurred for this system.

**Figure 5:**
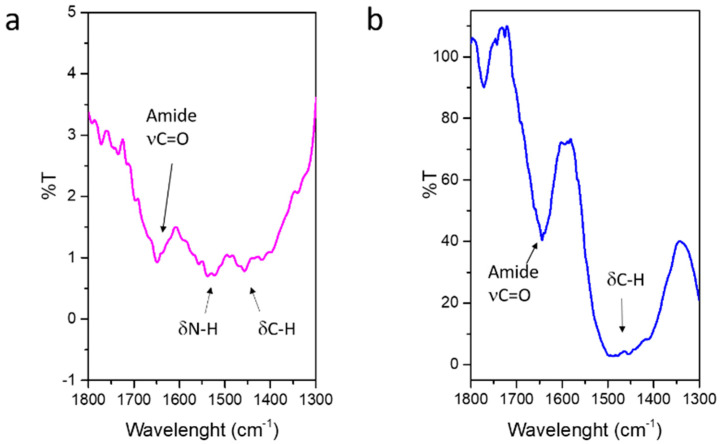
Difference FT-IR spectra of SG-Sr-ICOS-Fc (**a**) SD-Sr-ICOS-Fc (**b**); the spectra of SG-Sr and SD-Sr have been subtracted, respectively.

The successful ICOS-Fc binding to MBG surface has been investigated by FT-IR spectroscopy (Figure S2). Figure 5 reports the difference spectra in the range of 1800–1300 cm^−1^ of SG-Sr-ICOS-Fc and SD-Sr-ICOS-Fc obtained by subtracting the spectra of SG-Sr and SD-Sr, respectively. The signals due to the stretching of amide carbonyl group (C=O) is observed in the range 1680–1650 cm^−1^ (free and H-bonded amides), while at lower frequencies the C-H and N-H bending modes of amine functionalities and ICOS-Fc structure are visible. It is worth to note that for SG-Sr-ICOS-Fc the signal due to N-H bending is more intense compared to the SD-Sr-ICOS-Fc, suggesting a higher number of residual amines (not reacted with ICOS-Fc), most likely due to the smaller pore size and the consequent limited reactivity at the pore entrances.

### 3.2. In Vitro Bioactivity of SG-Sr-ICOS-Fc and SD-Sr-ICOS-Fc

Since the developed materials are supposed to preserve the ability to promote hydroxyapatite deposition when in contact with physiological fluids, the bioactive behavior after ICOS-Fc functionalization was one of the major goals of the work. Moreover, the effect of strontium amount into MBG framework on HA formation remains an open issue in the literature. On the one hand, the incorporation of strontium is reported to increase the HA formation, due to the decrease of the silica network connectivity [34,35]. On the other hand, the substitution of calcium ions with strontium ions could decrease the amount of released calcium ions, leading to a reduction of the apatite-forming ability [31,36]. Moreover, the ion-exchange reactions, essential to promote the deposition of HA, could be compromised or even fully prevented by the presence of ICOS-Fc on MBG surface. According to the morphological and structural analysis performed by means of FE-SEM and XRD, the kinetics of HA formation of SG-Sr-ICOS-Fc and SD-Sr-ICOS-Fc appeared not significantly hampered by the presence of strontium within MBG framework and by the overall process of biofunctionalization, if compared to data previously obtained by the authors for both unsubstituted silica-calcium oxide MBG and Sr-substituted MBG containing a lower amount of strontium [16].

**Figure 6:**
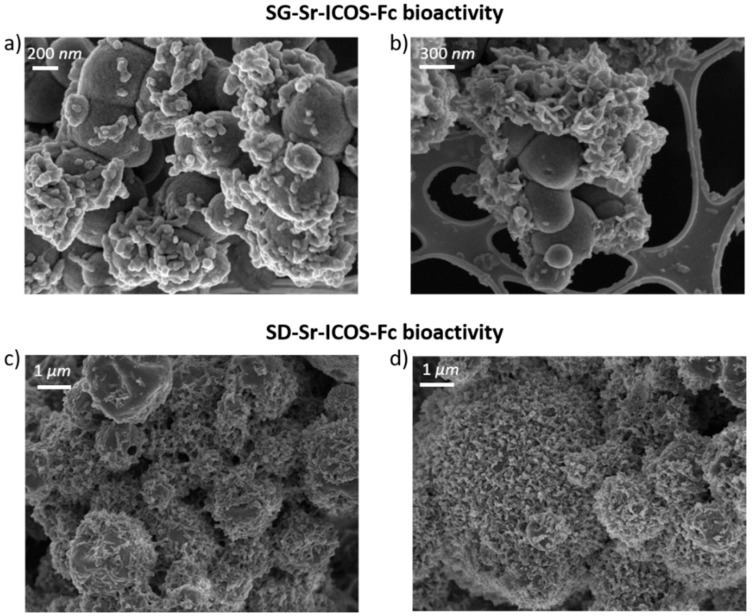
FESEM images of SG-Sr-ICOS-Fc bioactivity after 1 day (**a**) and 7 days (**b**) of soaking in SBF, SD-Sr-ICOS-Fc after 1 day (**c**) and 7 days (**d**) of soaking in SBF.

**Figure 7:**
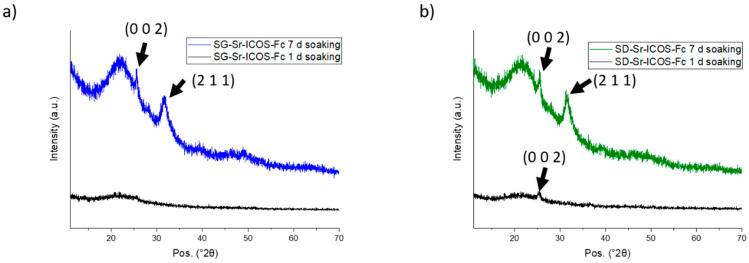
XRD at different time steps of soaking in SBF of SG-Sr-ICOS-Fc (**a**) and SD-Sr-ICOS-Fc (**b**).

SG-Sr-ICOS-Fc and SD-Sr-ICOS-Fc were analyzed and proved a considerable bioactivity, observed by their capacity to induce the deposition of hydroxyapatite with a fast kinetics [37]. In particular, FE-SEM images showed the deposition of an apatite-like phase characterized by the presence of rough layers on the MBG surface both for SG-Sr-ICOS-Fc and SD-Sr-ICOS-Fc, already after one day of soaking as shown in Figure 6a and 6c, respectively. After 7 days, MBG particles resulted fully covered by a compact layer of needle-like nanocrystals with the characteristic cauliflower morphology (Figure 6b,d). Moreover, EDS analysis on powder reported a Ca/P ratio of 1.7, similar to the value reported in the literature for the carbonated hydroxyapatite [16,38]. XRD analysis performed on SG-Sr-ICOS-Fc and SD-Sr-ICOS-Fc after soaking in SBF confirmed the formation of nanocrystalline hydroxyapatite layers by the formation of two peaks at 25.8 and 32.0° 2θ after 7 days (Figure 7) assigned to the (0 0 2) and (2 1 1) reflection of HA, respectively (reference code 01-074-0565) [16,39].

### 3.3. Sr^2+^ Ions Release from SG-Sr-ICOS-Fc and SD-Sr-ICOS-Fc

**Figure 8:**
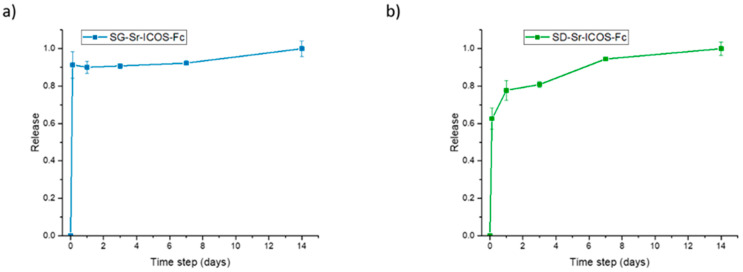
Strontium release profiles of SG-Sr-ICOS-Fc (**a**) and SD-Sr-ICOS-Fc (**b**).

The ion release of ICOS-grafted samples was evaluated in Tris-HCl medium at pH 7.4. The samples were incubated at 37 °C up to 14 days and, at predefined time points (3 h, 1 day, 3 days, 7 days, and 14 days) were centrifuged, aliquots were withdrawn and analyzed by ICP-AES. As shown in Figure 8, both SG-Sr-ICOS-Fc and SD-Sr-ICOS-Fc after ICOS grafting procedures maintained their ability to release the total amount of incorporated Sr^2+^ ions with a release kinetics comparable to those observed for analogue Sr-substituted MBGs [16]. In particular, SG-Sr-ICOS-Fc displayed a burst effect during the first 3 h which reaches a plateau phase after 1 day (Figure 8a), while SD-Sr-ICOS-Fc showed a more sustained release profile over time. The burst ion release observed from the SG samples, in fact, could be associated to the remarkably higher surface area and the lower particle size, in which the short diffusion paths allow faster ion diffusion inside the porous structure [40]. On the contrary, the sustained release profile of SD-Sr-ICOS-Fc could be ascribed by the progressive occlusion of pores due to the dissolution of the silica framework and its re-precipitation as silica gel at the pores mouth [41,42]; these phenomena can be explained by a less condensed framework, compared to SG-Sr-ICOS-Fc, due to the rapid evaporation of the solvent encountered during the aerosol-assisted spray-drying process. Moreover, irrespective of the MBG synthesis approaches, the ICOS grafting did not alter the ability to release the therapeutic ions, confirming the fast ion diffusion. The amount of strontium released by both the systems has been proved, as reported in the literature by Jones and co-workers [43,44], to stimulate the osteogenic response without inducing any cytotoxic effect. In fact, Sr-containing glasses with the composition of 90 mol% SiO_2_, 4.4 mol% CaO and 9.4 mol% SrO, thus very close to those reported in this work, were able to stimulate an osteogenic response from human bone marrow MSCs (hMSCs), even when the cells were cultured with only the particle extracts, indicating an effect specifically related to the release of Sr^2+^ and Ca^2+^ ions [23].

### 3.4. Detection of ICOS-Fc Grafted on SG-Sr-ICOS-Fc and SD-Sr-ICOS-Fc by ELISA-Like Assay

**Figure 9:**
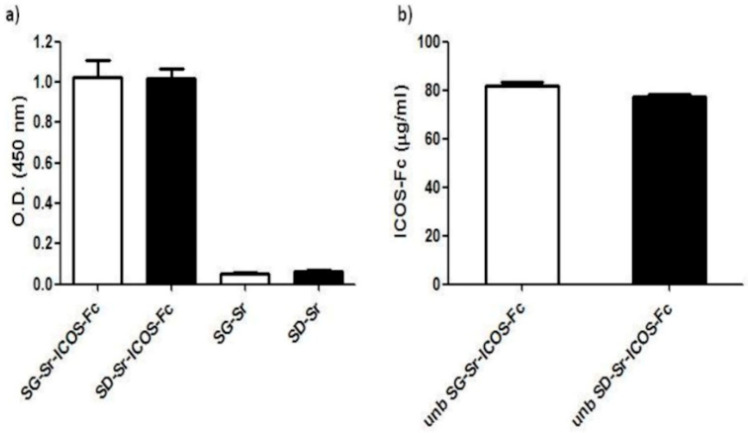
ELISA-like assay on (**a**) SG-Sr-ICOS-Fc, SD-Sr-ICOS-Fc, SG-Sr, SD-Sr, and (**b**) unb ICOS-Fc after grafting reaction. The binding and the amount of ICOS-Fc were detected by ELISA-like assay using the human ICOSL-His as the capture protein and anti-human-Hrp as detection antibody. Data are reported as Optical Density (OD) values and as concentrations expressed in μg/mL (mean and standard error were obtained from three independent samples evaluated in duplicate).

In order to check if ICOS-Fc grafted on the surface of MBGs maintained its ability to bind ICOS-L, (the ICOS binding partner), samples were tested by a home-made ELISA-like assay using recombinant ICOS-L to detect ICOS-Fc. The assay results, reported in Figure 9a, show that SG-Sr-ICOS-Fc and SD-Sr-ICOS-Fc gave higher OD values compared to those obtained with the reference blank samples (SG-Sr and SD-Sr respectively), suggesting that ICOS-Fc was successfully grafted and preserved its ability to bind ICOSL. Samples of SG-Sr and SD-Sr gave OD values comparable to the blank of the assay (OD value = 0.05), confirming no related specific signals in the ELISA-like assay (Figure 9a). The same assay was conducted to detect the unbounded (unb) ICOS-Fc contained in the supernatant recovered after MBG grafting with the dual purpose of: (i) assessing the potential alteration of the ICOS-Fc functionality caused by the reagents used for the grafting reaction and/or the overall reaction procedure and (ii) of calculating the amount of ICOS-Fc molecules grafted to MBGs by exploiting a subtractive calculation method.

The results showed that the amount of unb ICOS-Fc detected in the supernatants collected from both SG-Sr-ICOS-Fc and SD-Sr-ICOS-Fc suspensions resulted to be about 80 µg/mL (Figure 9b). This amount was subtracted from the initial amount of ICOS-Fc used during the grafting reaction (1 mg of ICOS-Fc in 10 mL of suspension containing 500 mg of MBG particles). Based on the number of particles and the volumes employed in the grafting reaction, the average ICOS-Fc grafted amount was calculated to be 0.4 µg per mg of MBG particles.

### 3.5. Detection of ICOS-Fc Grafted on SG-Sr-ICOS-Fc and SD-Sr-ICOS-Fc by Flow Cytometry

The presence of grafted ICOS-Fc was also confirmed by flow-cytometry, that enabled a more specific analysis on individual MBG particle. To this purpose, the latter were firstly stained with an APC-conjugated anti-ICOS monoclonal antibody able to bind the ICOS-Fc grafted on MBG particles and subsequently analyzed obtaining the MFI-R (mean fluorescence intensity ratio), which was calculated with the following equation:

$$\mathrm{MFI}-R=\;\frac{{\mathrm{MFI}}_{\left(\mathrm{stained}\;\mathrm{sample}\right)}}{{\mathrm{MFI}}_{\left(\mathrm{Control}\right)}}$$

**Figure 10:**
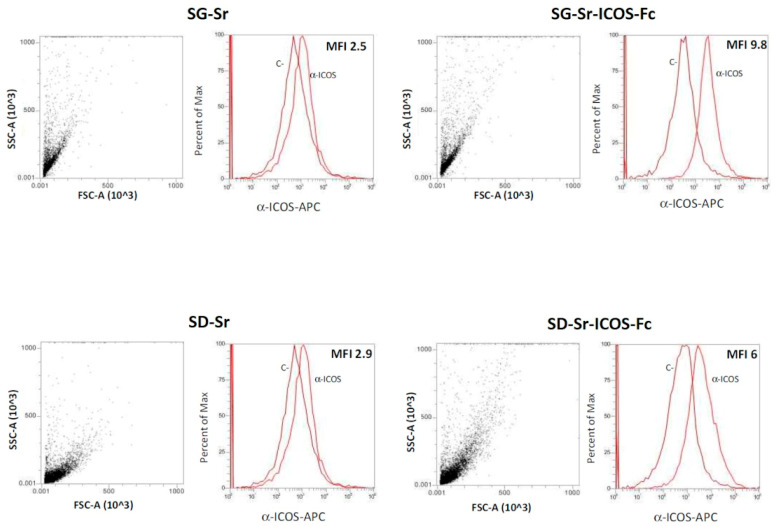
Cytofluorimetric analysis and MFI-R results for SG-Sr-ICOS-Fc, SD-Sr-ICOS-Fc, SG-Sr and SD-Sr. Dot plots and cytofluorimetric histograms of ICOS-Fc for each sample tested are shown. C-: unstained sample; α-ICOS: sample stained with the antibody. FSC: forward scatter; SSC: side scatter.

SG-Sr and SD-Sr were used as negative control. The results reported in Figure 10 further confirmed that ICOS-Fc was successfully grafted on SG-Sr-ICOS-Fc and SD-Sr-ICOS-Fc. In fact, higher values of MFI-R were detected for both SG-Sr-ICOS-Fc and SD-Sr-ICOS-Fc (9.8 and 6, respectively) due to the presence of ICOS-Fc grafted on MBG surface compared to those obtained for the bare samples (about 2.5 and 2.9 for SG-Sr and SD-Sr, respectively).

### 3.6. Detection of ICOS-Fc Grafted on SG-Sr-ICOS-Fc and SD-Sr-ICOS-Fc Post-Soaking in DMEM by ELISA-Like Assay

In order to evaluate ICOS-Fc binding stability to hydrolysis reaction in aqueous medium, SG-Sr-ICOS-Fc and SD-Sr-ICOS-Fc were soaked in DMEM for 3, 7, 14 and 21 days. The time points were specifically chosen to mimic the time-course of osteoclast differentiation in vitro, where monocytes are cultured for 21 days in a differentiation medium composed of DMEM, recombinant human M-CSF and RANK-L changing the differentiation medium every 3 days [22].

An analysis of the recovered supernatants using the ELISA-like assay with ICOSL revealed a minimum amount of ICOS-Fc in the medium both for SG-Sr-ICOS-Fc and SD-Sr-ICOS-Fc at each time point. The percentage of ICOS-Fc released during soaking was calculated comparing the concentration measured in the DMEM with the effective amount of grafted ICOS-Fc, corresponding to 0.4 µg/L mg of MBG, as previously described in Section 3.3. The related values at the different time steps are very low (Table 2) and suggest the formation of a stable covalent bond upon grafting reaction. The minimal quantity of detected ICOS-Fc in the supernatants is ascribable to adsorbed molecules weakly interacting through intermolecular interactions (H-bonding, dispersive forces) with residual MBG silanol groups.

**Figure 11:**
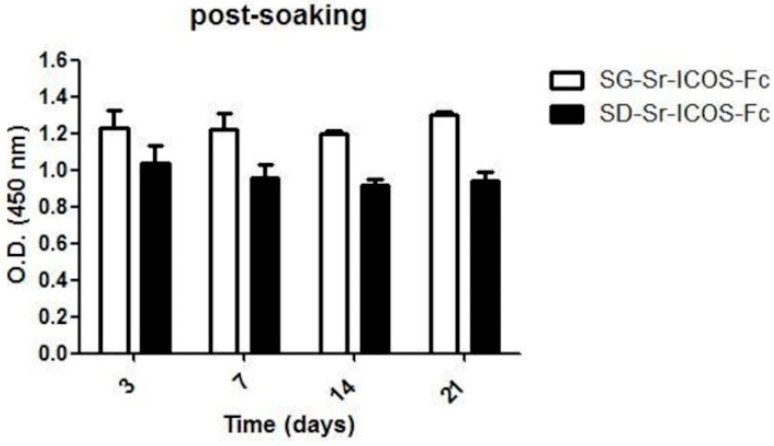
ELISA-like assay conducted on SG-Sr-ICOS-Fc and SD-Sr-ICOS-Fc post soaking in DMEM collected at different time steps (3, 7, 14 and 21 days). The presence and the binding of ICOS-Fc were detected by ELISA-like assay using the human ICOSL-His as capture protein and anti-human-Hrpas detection antibody. The graph shows the Optical Density (OD) values (mean and standard error were obtained from three separate samples).

Furthermore, as a further evidence of the binding stability, ELISA-like assay using ICOSL was carried out on SG-Sr-ICOS-Fc and SD-Sr-ICOS-Fc samples collected at different time steps (3, 7, 14, and 21 days) and confirmed that ICOS-Fc was still grafted on up to 21 days of soaking in DMEM (Figure 11).

### 3.7. Biocompatibility Assessment of SG-Sr-ICOS-Fc and SD-Sr-ICOS-Fc

**Figure 12:**
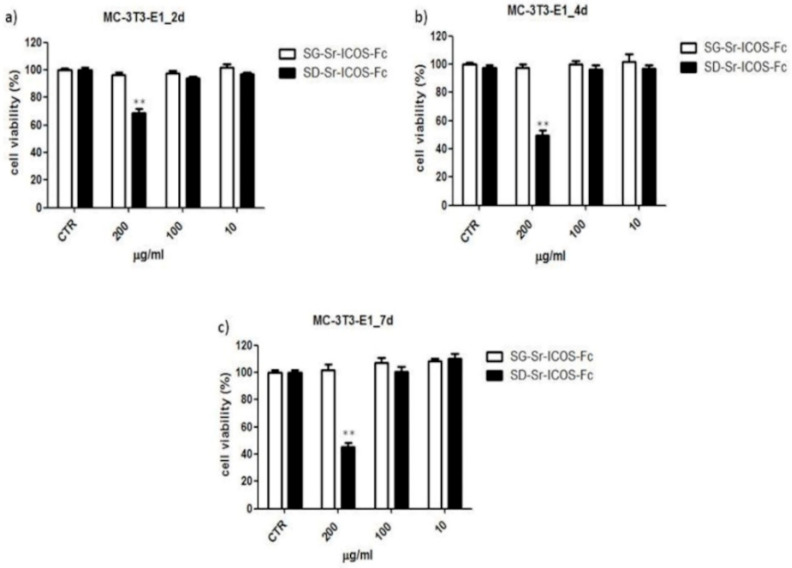
Cell viability studies performed on SG-Sr-ICOS-Fc (white bars) and SD-Sr-ICOS-Fc (black bars) samples with MC3T3-E1 cell line at different exposure times (**a**) 2 d, (**b**) 4 d, and (**c**) 7 d considering different particle concentrations. ** *p* < 0.01 vs. untreated cells (CTR) (Dunnett’ s test). The graphs show cell viability (%) as mean and standard error obtained from three independent experiments. Cell viability was calculated with the following formula: cell viability = absorbance of sample/absorbance of control (untreated cells) × 100.

To evaluate cytotoxicity, MC3T3-E1 murine pre-osteoblast cells were incubated with different amounts of both SG-Sr-ICOS-Fc and SD-Sr-ICOS-Fc (200–100–10 μg/mL). At defined incubation times (2, 4 and 7 days) cell viability was evaluated by a standard cell viability assay using XTT reagent. Results showed a cell viability ranging from 70% to 45% in presence of SD-Sr-ICOS-Fc at the highest concentration tested (200 μg/mL). The other tested concentrations did not show significant modifications in cell viability compared to untreated cells. On the contrary, SG-Sr-ICOS-Fc did not affect cell viability, showing results comparable to untreated cells (Figure 12).

### 3.8. Effect of SG-Sr-ICOS-Fc and SD-Sr-ICOS-Fc on Cell Migration and Proliferation

Since the effect of free ICOS-Fc on cell migratory activity was reported by the authors [44,45], the retention of this essential biological effect was also assessed for grafted ICOS-Fc, by using the Boyden chamber migration assay both with ICOSL positive cell lines, PC-3 (prostate cancer) and U2OS (osteosarcoma), or ICOSL negative cell line, HOS. Cells were suspended in serum-free medium, seeded in the upper chamber in the presence of increasing concentrations (in the 0.002–2 μg/mL range) of SG-Sr-ICOS-Fc or SD-Sr-ICOS-Fc and allowed to migrate for 6 h towards the lower chamber containing medium +20% FCS, used as migration stimulus. The same experiment was conducted by using free ICOS-Fc molecule and SG-Sr and SD-Sr as blank samples.

**Figure 13:**
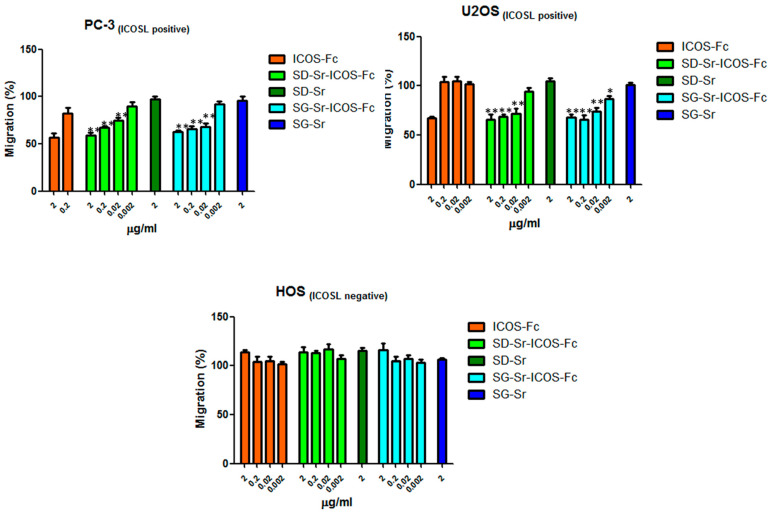
Cells were plated onto the apical side of Matrigel-coated filters in the presence and absence of either SD-Sr-ICOS-Fc, SG-Sr-ICOS-Fc, SD-Sr, and SG-Sr. ICOS-Fc was used as positive control. FBS 20% was loaded in the basolateral chamber as a chemotactic stimulus. Data are expressed as mean± SEM (*n* = 5) of the percentage of migration versus control migration (FBS 20%). * *p* < 0.05; ** *p* < 0.01 SG-Sr-ICOS-Fc vs. SG-Sr and SD-Sr-ICOS-Fc vs. SD-Sr.

The results reported in Figure 13 show that PC-3 and U2OS cell migration was affected in a dose dependent manner by SG-Sr-ICOS-Fc or SD-Sr-ICOS-Fc, evidencing the retained ability of ICOS-Fc when grafted to inhibit the cell motility. By contrast, HOS (ICOSL negative) cell migration was not affected by SD-Sr-ICOS-Fc or SG-Sr-ICOS-Fc or free ICOS-Fc, confirming the specificity of ICOS-Fc to bind ICOSL. As expected, SG-Sr and SD-Sr did not show any effect on cell migration. Furthermore, the incubation with SD-Sr-ICOS-Fc or SG-Sr-ICOS-Fc, as well as the blank samples, for 6 h did not show cell cytotoxicity, evaluated by crystal violet (data not shown).

**Figure 14:**
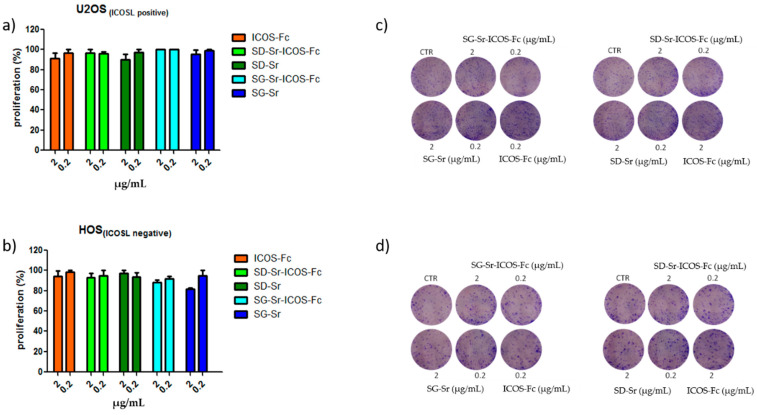
Clonogenic assay: (**a**) U2OS; (**b**) HOS. Cells were treated with ICOS-Fc, SD-Sr-ICOS-Fc, SD-Sr, SG-Sr-ICOS-Fc, and SG-Sr at 2 and 0.2 μg/mL concentration for 72 h. Then, the cell medium was changed, and the cells were cultured for additional 7 days in a free medium (**c**), (**d**) Colonies were then photographed. Then, the cells were treated with acetic acid to induce a completely dissolution of the crystal violet and absorbance was evaluated. Three different experiments were performed. Data are shown as mean ± SEM.

In order to investigate the effect of SG-Sr-ICOS-Fc and SD-Sr-ICOS-Fc on cell proliferation, clonogenic proliferation assays were performed. Related results reported in Figure 14 demonstrated that SG-Sr-ICOS-Fc, SD-Sr-ICOS-Fc, SG-Sr, and SD-Sr did not affect cell proliferation.

### 3.9. Effect of SG-Sr-ICOS-Fc and SD-Sr-ICOS-Fc on Monocyte-Derived Osteoclasts (MDOC) Differentiation

**Figure 15:**
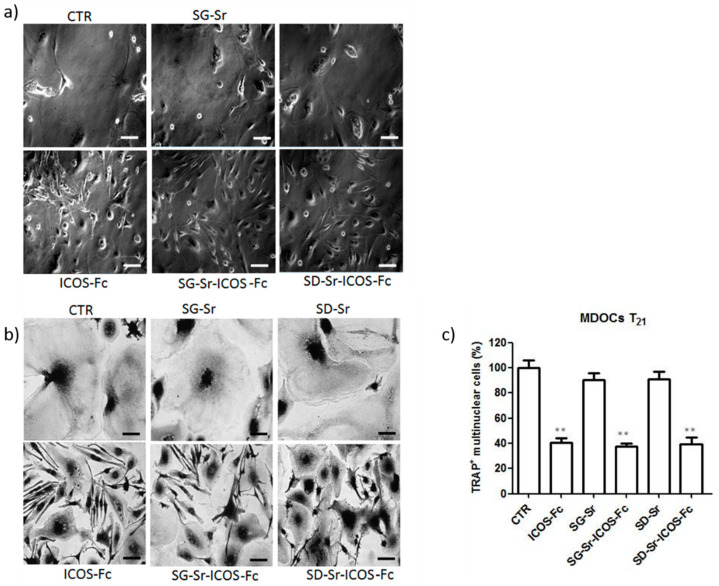
Effect of ICOS-grafted MBGs on MDOC differentiation using the T0–21 treatments. Monocytes were induced to differentiate to MDOCs in the presence and absence of SG-Sr-ICOS-Fc and SD-Sr-ICOS-Fc from day 0 (T0–21 treatment). (**a**) Phase-contrast microscopy of cells at T21 observed at X20 original magnification (**b**) Microphotographs of TRAP staining at T21 were observed at original magnification X20. (**c**) Bar graphs show the percentage of the multinuclear TRAP+ cells at T21. Data are expressed as the mean± SEM of the percentage of inhibition versus the control (set at 100%) by counting 10 fields per sample (** *p* < 0.01 versus the control; Dunnett’s test). Scale bar 125 μm.

The inhibitory effect of free ICOS-Fc on the differentiation of monocyte-derived osteoclasts (MDOCs) was reported by the authors [22] and the retention of this biological property by ICOS-Fc grafted on MBG surface is essential. Therefore, MDOCs were contacted with SG-Sr-ICOS-Fc and SD-Sr-ICOS-Fc and the differentiation process was monitored up to 21 days (T21). The obtained results reported in Figure 15 show that T_0–21_ treatments with MBGs exposing ICOS-Fc were able to strongly inhibit MDOC differentiation, in analogy to free ICOS-Fc. After the treatments with SG-Sr-ICOS-Fc and SD-Sr-ICOS-Fc, the cells showed a round shape and a morphology similar to a spindle, comparable to the effect exerted by free ICOS-Fc. By contrasts, SG-Sr and SD-Sr did not show any effect on MDOC differentiation and the morphology was similar to untreated MDOCs. Moreover, treatments with SG-Sr-ICOS-Fc and SD-Sr-ICOS-Fc showed a decreased formation of multinuclear TRAP^+^ cells, expressed as percentage of TRAP^+^ cells.

**Figure 16:**
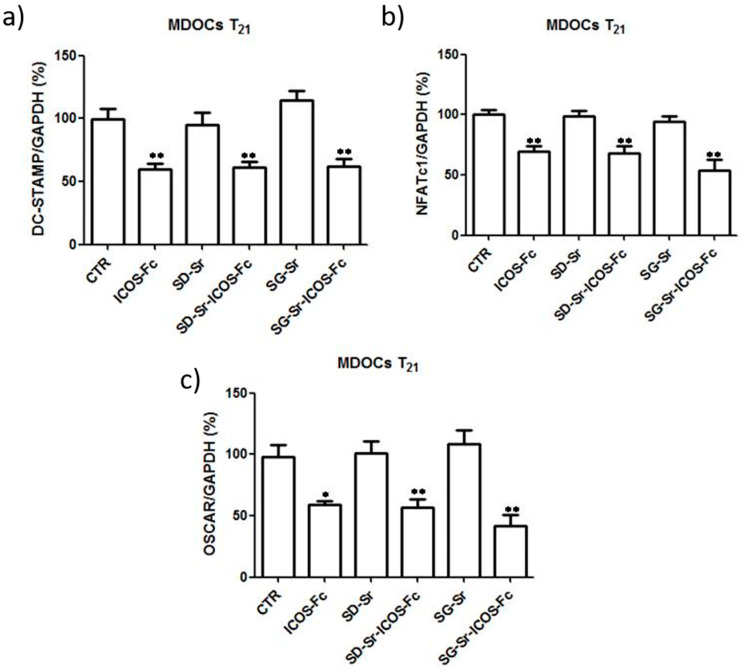
Effect of MBGs reagents on MDOC expression of DC-STAMP, NFATc1, and OSCAR. Bar graphs show the real-time PCR data of expression of (**a**) DC-STAMP, (**b**) NFATc1, and (**c**) DC-STAMP at T21. Data are expressed as the mean ± SEM. The data are normalized for the expression in the control cells (control expression set at 100%). * *p* < 0.05, ** *p* < 0.01 versus the control (Dunnett’s test).

Since osteoclast differentiation is marked by the upregulation of DC-STAMP, OSCAR, and NFATc1 expression, the effect of grafted ICOS-Fc samples on the expression of these genes by real-time PCR at T21 was evaluated. The results reported in Figure 16 showed that SG-Sr-ICOS-Fc and SD-Sr-ICOS-Fc significantly decreased the expression of all these mRNAs compared with the untreated cells and cells treated with SD-Sr and SG-Sr.

## 4. Conclusions

The presented study reports the successful development of a novel multifunctional nano-biomaterial able to stimulate bone regeneration in critical clinical situations (i.e., osteoporotic fractures) through the combination of chemical (ion release) and biological cues. In particular, ICOS-Fc molecule, chosen for its ability to reversibly inhibit osteoclast differentiation and activity, was effectively grafted to the surface of Sr-containing MBGs, in order to combine the anti-osteoclastic effect provided by the anchored biomolecules with the pro-osteogenic properties exerted by released ions (silicate, calcium and strontium ions). To this aim Sr-containing MBGs (10 mol%) in the form or micro and nanoparticles were obtained by two different synthesis methods and successively functionalized in order to expose reactive amino groups able to form a stable amide bond with ICOS-Fc molecule. The evident decrease of the specific surface area and pore volume of MBGs after the two-step functionalization, the amide bond formation evidenced by FT-IR spectroscopy and results from flow cytometry assays proved the effective grafting of ICOS-Fc molecule on Sr-containing MBGs. The functionality of the anchored ICOS-Fc molecules (as the ability to bind its ligand ICOSL) and their binding stability was assessed by a custom-made ELISA-like, which demonstrated a full retention of functionality and a negligible loss of ICOS-Fc up to 21 days of soaking in culture medium. MC3T3-E1 murine pre-osteoblast cells showed high viability even when contacted with high concentrations of the functionalized MBG particles, confirming their excellent cytocompatibility and the inhibitory effect of grafted ICOS-Fc on cell migratory activity was demonstrated by using ICOSL positive cell lines, PC-3 (prostate cancer) and U2OS (osteosarcoma). At variance, the same experiment carried out with ICOSL negative cell line (HOS) did not reveal any inhibitory effect, suggesting the specificity of ICOS-Fc to bind its ligand ICOSL. Finally, the ability to inhibit osteoclast differentiation and function was confirmed for grafted ICOS-Fc (in analogy to the free form of the molecule) by monitoring the differentiation of monocyte-derived osteoclasts (MDOCs), which revealed a strong inhibitory effect, also proved by the downregulation of osteoclast differentiation genes.

The obtained results are very promising and pave the way to further in vivo studies in order to implement multifunctional and personalized clinical solutions for the bone regeneration of fractures in osteoporotic patients.

## Figures and Tables

**Table 1:** SSA_BET_ and total pore volume of SG-Sr, SD-Sr, SG-Sr-ICOS-Fc, and SD-Sr-ICOS-Fc.

Sample	SSA_BET_ (m^2^/g)	Pore Volume (cm^3^/g)	Pore Size (nm)
SG-Sr	465	0.33	4
SD-Sr	85	0.23	7–11
SG-Sr-ICOS-Fc	34	0.09	N/A
SD-Sr-ICOS-Fc	13	0.03	N/A

**Table 2:** Stability test of ICOS-Fc binding to MBG surface. The amount of ICOS-Fc released was evaluated for both SG-Sr-ICOS-Fc and SD-Sr-ICOS-Fc at time steps of 3, 7, 14 and 21 days.

Sample	Time Step (Days)	ICOS-Fc Percentage Released (%)	Standard Deviations
SG-Sr-ICOS-Fc	3	4.2	2.3
	7	5.4	2.4
	14	5.6	0.9
	21	6.7	0.3
SD-Sr-ICOS-Fc	3	5.6	0.3
	7	5.3	1.4
	14	5.4	0.8
	21	6.6	0.7

## Data Availability

The data presented in this study are openly available in ZENODO at 10.5281/zenodo.4074877; 10.5281/zenodo.4074881; 10.5281/zenodo.4074883; 10.5281/zenodo.4468024.

## Supplementary Materials

The following are available online at https://www.mdpi.com/2079-4991/11/2/321/s1. The original contributions presented in the study are publicly available. These data can be found here: [10.5281/zenodo.4055360; 10.5281/zenodo.4074878; 10.5281/zenodo.4074882; 10.5281/zenodo.4074884].
