# Supplementary material for: Sr-Containing Mesoporous Bioactive Glasses Bio-Functionalized with Recombinant ICOS-Fc: An In Vitro Study
Source: Nanomaterials (Basel). 2021 Jan 27;11(2):321. doi: 10.3390/nano11020321 (PMC7911784; doi:10.3390/nano11020321)
Supplement: Supplementary file 1 [file nanomaterials-11-00321-s001.pdf]

nanomaterials

MDPI

Supporting information

Sr-Containing Mesoporous Bioactive Glasses Bio-Functionalized with Recombinant ICOS-Fc: An In Vitro Study

Sonia Fiorilli [1,*], Mattia Pagani [1], Elena Boggio [2,3], Casimiro Luca Gigliotti [2,3], Chiara Dianzani [4], Rémy Gauthier [1], Carlotta Pontremoli [1], Giorgia Montalbano [1], Umberto Dianzani [3] and Chiara Vitale-Brovarone [1]

Table S1. EDS analysis of SG-Sr-ICOS-Fc (a) and SD-Sr-ICOS-Fc (b).

a)

Element	Wt%	Wt% Sigma
O	57.86	0.62
Si	31.81	0.46
Ca	1.89	0.16
Sr	8.43	0.64
Total:	100.00	

b)

Element	Wt%	Wt% Sigma
O	50.63	0.53
Si	35.18	0.41
Ca	3.09	0.15
Sr	11.09	0.54
Total:	100.00	

EDS analysis of SG-Sr-ICOS-Fc and SD-Sr-ICOS-Fc were performed by Field-Emission Scanning Electron Microscopy (FE-SEM) using a ZEISS MERLIN instrument. As reported in table S1, the grafting of ICOS-Fc molecule did not induce any loss of incorporated strontium amount.

However, the amount of incorporated strontium cannot be properly detected by this type of analysis due to the overlapping of the strontium and silicon signals.

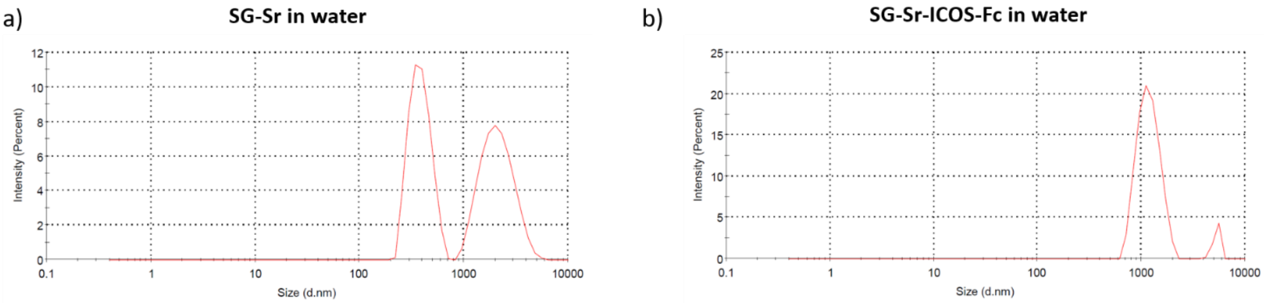

Figure S1. DLS analysis of SG-Sr in water (**a**) and SG-Sr-ICOS-Fc in water (**b**).

DLS analysis were performed with a Zetasizer nano ZS90 Malvern Instruments Ltd. Briefly, 3 mg of SG-Sr or SG-Sr-ICOS-Fc were dispersed in 3 mL of ddH_2O to obtain a concentration of 1 mg/mL and sonicated for 10 min. Then, the suspension was analysed.

Results related to SG-Sr showed a bimodal distribution with a maximum at around 390 nm size (in good accordance with morphological analysis) and a second one at around 2200 nm size, attributed of MBGs aggregates. The formation of aggregates in aqueous medium is very common and expected for these particles when a dispersing agent (i.e. surfactant) is not employed. Microsized aggregates not significantly hinder the properties of the final materials in term of ion release or bioactivity since the latter is strictly dependent on the internal surface area of the material. For SG-Sr-ICOS size the instrument displayed 1200 nm size and 5300 nm size values. In fact, the presence of a biomolecule on the MBGs surface is expected to increase the diameter by creating a large hydration sphere ascribable to the ability of the biomolecule to binds water molecules.

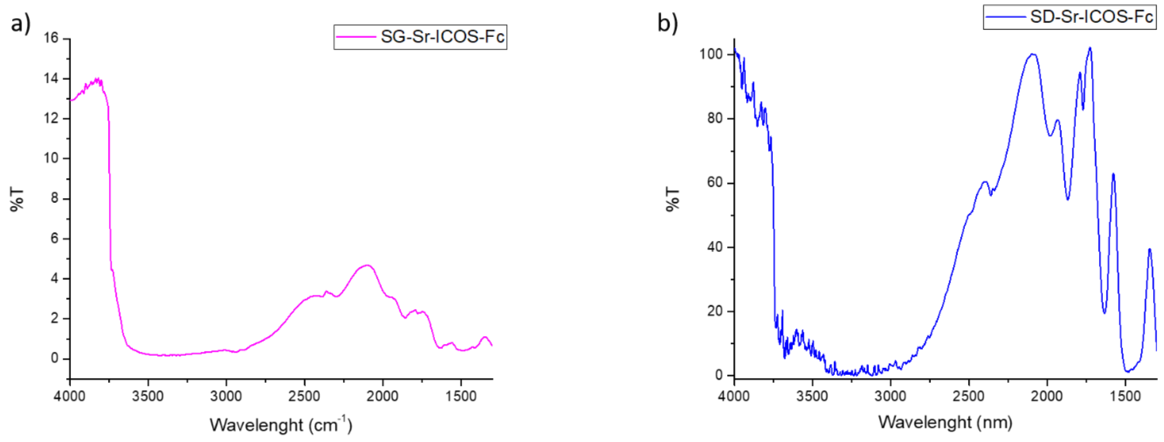

Figure S2. Full-range FT-IR spectra of SG-Sr-ICOS-Fc (**a**) and SD-Sr-ICOS-Fc (**b**). Spectra were plotted in % of Transmittance.

Full-range FT-IR spectra of SG-Sr-ICOS-Fc and SD-Sr-ICOS-Fc plotted in % of transmittance. The broad peak in the 3000-3500 cm^{-1} is due to the large amount of water bonded to the ICOS-Fc molecule. However, the range between 1800-1300 cm^{-1} is the most representative to prove the anchoring of ICOS-Fc molecule.
